# Over-Expression of *Cyclooxygenase-2* in Colorectal Cancer Patients

**DOI:** 10.31557/APJCP.2019.20.6.1675

**Published:** 2019

**Authors:** Ram Rattan Negi, Satya Vati Rana, Vikas Gupta, Rajesh Gupta, Vijayta Dani Chadha, Kaushal Kishor Prasad, Devinder K Dhawan

**Affiliations:** 1 *Department of Biophysics, *; 4 *Centre for Nuclear Medicine, Panjab University, *; 2 *Department of Gastroenterology, *; 3 *Department of General Surgery, Postgraduate Institute of Medical Education and Research (PGIMER), Chandigarh, India. *

**Keywords:** Cyclooxygenase-2- real-time polymerase chain reaction- gene expression- colorectal cancer

## Abstract

**Background:**

Colorectal carcinoma (CRC) is the most common neoplasm of the gastrointestinal tract. COX-2 plays an important role in CRC development and is a key target for the regression of colorectal tumorigenesis by non-steroidal anti-inflammatory drugs. The present study was conducted to examine the relationship of the levels of COX-2 in CRC patients with the clinico-pathological parameters and also to assess its usefulness as a potential biomarker for diagnosis of CRC.

**Methods:**

Prior to surgery, 30 CRC patients were enrolled and the samples from colon tumors and surrounding tissues were taken after they underwent surgical intervention at PGIMER, Chandigarh. mRNA expression levels of COX-2 were examined in 30 CRC and adjacent normal colonic mucosa by quantitative polymerase chain reaction (qPCR). The expression of COX-2 was assessed by immunohistochemical method using rabbit polyclonal antibodies against human COX-2 protein.

**Results:**

The quantitative relative expression of COX-2 mRNA was observed to be significantly higher (p<0.05) in colorectal cancer tissues as compared to adjacent normal colon tissues. Also, female CRC patients showed significantly higher (p<0.009) expression of COX-2 mRNA vis-a-vis male colorectal cancer patients. This is the ﬁrst study which has reported a direct relationship between COX-2 mRNA expressions in male colorectal cancer patients versus females. Further, immunohistochemistry of COX-2 confirmed the quantitative real time-PCR findings.

**Conclusion:**

Our study shows that COX-2 over expression in colorectal carcinoma patients is closely associated with clinico-pathological parameters and is more pronounced in males versus females. Further, COX-2 mRNA expression can serve as a potential biomarker for the diagnosis of CRC.

## Introduction

Colorectal cancer (CRC) is a very common malignant tumor of the digestive tract. Nearly 1.2 million new cases of CRC and 600,000 associated deaths are reported worldwide annually (Wu and Sun, 2015; CRC is the third most common cancer in the world and the second most common cause of cancer-related deaths (Ricchi et al., 2003). In India, the annual incidence rates (AARs) for colon cancer and rectal cancer in men are 4.4 and 4.1 per 100,000, respectively. Whereas, AAR for colon cancer in women is 3.9 per 100,000. Colon cancer ranks 8th and rectal cancer ranks 9th among men in India (NCRP, 2013).The development of colorectal cancer is a highly complex and multistep process and is linked to mutations that may occur in the oncogenes K-ras and APC or in the tumor suppressor gene p53, thereby causing uncontrolled proliferation of cells.

Cell proliferation is pivotal in tumorigenesis and Cyclooxygenases (COXs) are important regulatory enzymes in this process. Cyclooxygenases catalyze the conversion of free arachidonic acid into prostaglandin H2, which is the precursor of other prostaglandins and thromboxanes. These regulatory compounds play a critical role in various biological processes such as cell proliferation, angiogenesis, immune function and inflammation, which are all crucial in the development as well as progression of neoplasms (Chandrasekharan et al., 2002).

The human COX family consists of three members, namely COX 1–3. COX-2 is an inducible enzyme, whose expression can be affected by pro-inflammatory and mitogenic stimuli like cytokines and growth factors. COX-2 plays an important role in the development of metaplastic and dysplastic tissues and also in the development and progression of cancer by its involvement in the regulation of cell proliferation, cell transformation, tumor growth, tumor metastasis and invasion (Chandrasekharan et al., 2002; Chandrasekharan and Simmons, 2004).

Previous studies have indicated that an increase in the expression of COX-2 has been associated with various premalignant and malignant lesions of epithelial origin, in particular in different regions of the gastrointestinal tract (Sano et al., 1995; Eberhart et al., 1994; Fujimura et al., 2006; Brown and DuBois, 2005; Mehta et al., 2006; Liu et al., 2008). Tumors with high levels of COX-2 seem to be more aggressive (Fujimura et al., 2006) and patients bearing those tumors had a significantly reduced survival (Buskens et al., 2002). COX-2 is of particular interest since specific inhibitors of COX-2 such as various non-steroidal anti-inflammatory drugs (NSAIDs) have been or are being developed that could have a role in the chemoprevention of gastrointestinal neoplasms (Brown and DuBois, 2005). Evidence is accumulating that a considerable reduction in the development of adenomatous polyps or colorectal cancer could be achieved in individuals regularly taking NSAIDs (Brown and DuBois, 2005). Although, most NSAIDs do not specifically inhibit COX-2 but the findings of over-expression of COX-2 in many pre-malignant tissues in combination with the reported beneficial effects of NSAIDs on cancer prevention do suggest that inhibition of COX-2 may be critical link in this process. Since, alterations in COX-2 levels may be essential in influencing the development of colorectal cancer, (Greenhough et al., 2009) so it is important that COX-2 levels can be evaluated in a reliable way.

Earlier, studies have reported that COX-2 expression is unregulated in precancerous lesions and preinvasive carcinoma and positively correlates with tumor invasion and lymphatic metastasis (Lurje et al., 2007; Strazisar et al., 2009). Therefore, increasing expression of COX-2 might occur in the early stages of the tumor and the detection of COX-2 level is helpful for early diagnosis of CRC. 

Thus, the aim of the present study was to quantify the COX-2 mRNA levels in colorectal tumor tissue and to assess the usefulness of mRNA expression as a potential marker for the diagnosis of colorectal cancer.

## Materials and Methods

*Subjects*

To carry out the present study patients from North Indian population prior to surgery were enrolled. Tumor tissue samples were taken from 30 patients of colorectal cancer after surgical intervention at general surgery operation theatre (OT) of PGIMER, Chandigarh. Unaffected colonic mucosa specimens were also collected in parallel with the tumor specimens from all the patients and served as normal control tissue. Immediately after resection, the tissue samples were transported on ice to the laboratory and the tissue samples were washed with ice cold phosphate buffered saline (PBS) and snap frozen in liquid nitrogen and stored at -80^o^C until use. The patients’ information related to smokers/non-smokers/alcohol consumption/family history and clinical history was recorded. Each patient was explained about the nature of the study and a written informed consent was obtained. The present study was approved by institutional ethics committee. Patients with confirmed diagnosis of colorectal cancer as reported by histopathologist were included. Patients with neuroendocrine carcinoma, malignant melanoma, non-Hodgkin’s lymphoma and gastrointestinal stromal tumors or patients suffering from other tumors along with CRC were excluded from the study.

*Total RNA Extraction and Reverse Transcription*

Total RNA was isolated from tumor and normal adjacent colonic mucosa using TRIzol reagent according to the manufacturer’s instructions (Invitrogen, USA). A total of 1 µg RNA was treated with 1 U DNase 1 (Fermentas) for 30 min at 37^o^C and then heat inactivated at 65^o^C for 10 min before reverse transcription to eliminate genomic DNA contamination.

cDNA was synthesized by reverse transcription from 1 µg RNA using Thermo Scientific’s RevertAid First Strand cDNA Synthesis Kit (Invitrogen) following manufacturer’s instructions in a total volume of 20µl.

*Quantitative Real Time PCR*

Real-time PCR analysis was performed on 96 Real-Time PCR system (Roche, Indianapolis, IN) using the SYBR Green PCR Master Mix (Roche Indianapolis, IN). The PCR reaction mixture consisted of 1x SYBR green reagent (Roche), 10 picomoles of each forward and reverse primers including 1 μl cDNA in a final volume of 10 μl reaction. Cycling conditions were 50°C for 10 seconds and 95°C for 10 minutes, followed by 40 cycles at 95°C for 15 seconds and 60°C for 1 minute. β-actin was used as an internal control.

The primers used in the experiment were as follows:

COX-2: Forward: 5′- CAGCCATACAGCAAATCC -3′; 

COX-2: Reverse: 5′-TCGCACTTATACTGGTCAA-3′ and β-actin: Forward: 5′- TCTACAATGAGCTGCGTG -3′; 

β-actin: Reverse: 5′- CCTTAATGTCACGCACGA- 3′. 

Specificity of the PCR products of COX-2 and β-actin was confirmed by melting curve analysis and agarose gel electrophoresis. All Real time qPCR analyses were performed in duplicates. Relative quantification of the expression of each gene was calculated by the 2^-Δ(Δct)^ method (Gibson et al., 1996).

*Immunohistochemistry*

The Avidin biotin complex (Universal) method was used for IHC staining. The antibody used was anti-COX2/Cyclooxygenase-2 antibody (Abcam laboratories) to detect COX-2 protein. Scoring of IHC was done by the scoring system. A score of 0-3 for staining intensity was assigned. An intensity score of 0= no staining, 1= weak positivity, 2= moderate positivity and 3= strong positive was recorded. Immunostaining was graded semi-quantitatively as negative, weak, moderate or strong corresponding to positive staining of >10, 10-25%, 25-50%, 50-100% of the total cells. The final IHC score was calculated as: IHC score= % age of positivity × intensity score.

*Statistical Analysis*

SPSS 20.0 software was used to perform statistical analysis. Statistical significance in COX-2 mRNA expression between two groups was evaluated by using one sample t-test and Mann–Whitney test. Whereas, significance among more than two groups was evaluated by using Kruskal -Wallis test. P values of less than 0.05 were considered statistically significant. All the results are represented as mean±standard error (S.E.).

## Results

*Patients’ Characteristics*

Out of 30 patients of colorectal cancer, 19 were males and 11 females. The patients were in the age group of 19-75 years with mean age of 51.7 ±14.3 years. Most of the cases were males (63%). Seventy percent of the cases were early (I-II) and 30% were advanced (III-IV) tumor stage. Most of the cases were moderately differentiated adenocarcinomas (86%). Similar number of cases of well differentiated (7%) and poorly differentiated adenocarcinomas (7%) was seen. The detailed clinical characteristics of patients are summarized in Table 1. 

**Table 1:** Clinical Characteristic of the Patients with Colorectal Cancer

Clinical Characteristics	N (%)
Patients (n)	30
Age	
≤50 years	14 (47%)
>50 years	16 (53%)
Sex	
Male	19 (63%)
Female	11 (37%)
Tumor Location	
Proximal	16 (53%)
Distal	14 (47%)
Clinical Stage (TNM)	
Early (I-II)	21 (70%)
Advanced (III-IV)	9 (30%)
Histopathological Type	
Adenocarcinoma	22 (73%)
Other Types	8 (27%)
Histopathological grade	
Well differentiated	2 (7%)
Moderately differentiated	26 (86%)
Poorly differentiated	2 (7%)

**Table 2:** Relationship between COX-2 mRNA Expression and Clinicopathological Features of Colorectal Cancer Patients

Clinicopathological parameters	Number of Cases (%)	RQ (Mean±SEM)	p-value
Age group			
≤50 years	14 (47%)	25.54± 8.14	0.249
>50 years	16 (53%)	14.07± 4.62	
Sex			
Male	19 (63%)	10.05± 3.73	0.009*
Female	11 (37%)	39.65± 8.73	
Tumor Location			
Proximal	16 (53%)	20.16± 6.42	0.68
Distal	14 (47%)	19.56± 6.81	
TNM stage			0.585
I + II	21 (70%)	21.46± 5.52	
III	9 (30%)	16.26± 8.90	
Lymph node metastasis			
Yes	9 (30%)	16.26± 8.90	0.585
No	21 (70%)	21.46 ± 5.51	
Histopathological Type			
Adenocarcinoma	22 (73%)	22.56± 5.62	0.132
Other Types	8 (27%)	13.65 ± 8.18	
Histopathological grade			
Good + Moderate	28 (93%)	18.59± 4.56	0.368
Poor	2 (7%)	36.58± 31.07	

COX-2, Cyclooxygenase-2; SEM, Standard error of the mean; TNM, Tumor Node Metastasis; RQ relative quantification *p<0.05, statistically significant difference

*Relationship between COX-2 mRNA Expression and Clinicopathological Features of Colorectal Cancer Patients*

**Figure 1:**
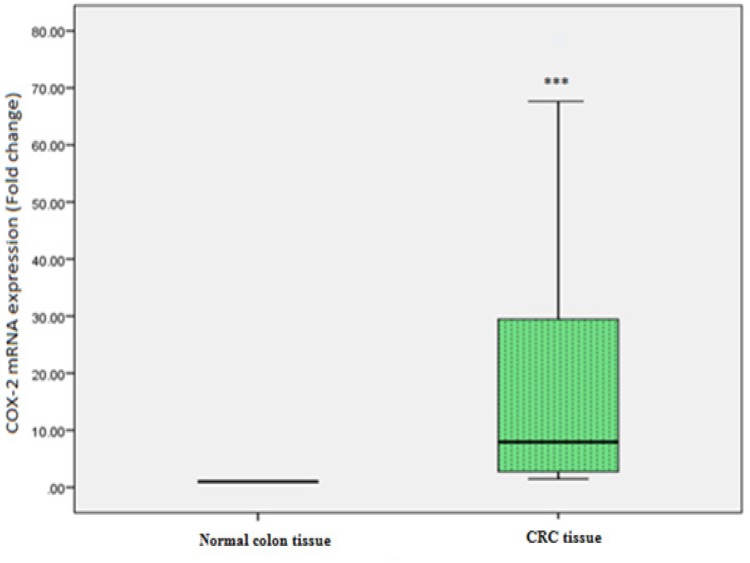
Cyclooxygenase-2 (COX-2) Gene Expression in Colorectal Carcinoma (CRC) and Adjacent Normal Colon Tissues. COX-2 mRNA levels in CRC as well as adjacent normal colon tissues were determined by real time PCR. β-actin mRNA levels were used to normalize COX-2 mRNA expression. Statistical analysis was done by means of one sample t-test. ***p < 0.001

**Figure 2:**
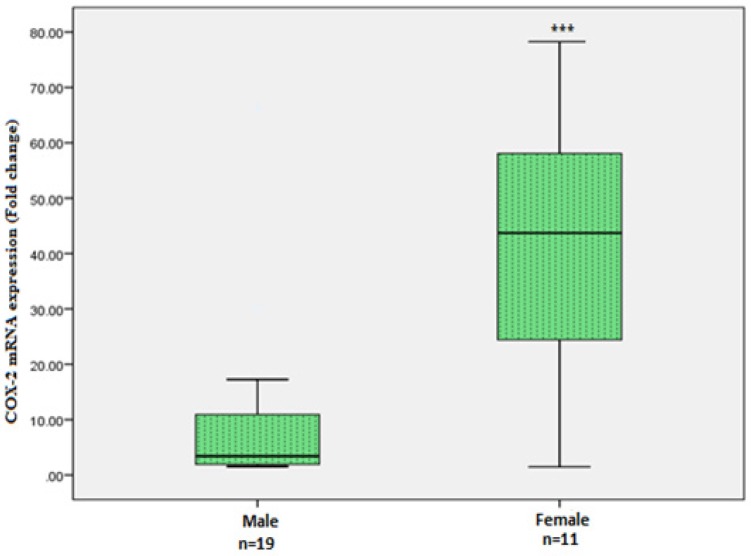
Cyclooxygenase-2 (COX-2) Gene Expression in Colorectal Carcinoma (CRC) Tissues of Males and Females. COX-2 mRNA levels in males and females of CRC tissues were determined by real time PCR. β-actin mRNA levels were used to normalize COX-2 mRNA expression. Statistical analysis was done by means of Mann–Whitney test. ***p < 0.01

The associations between COX-2 expression and clinicopathological features are presented in Table 2.The quantitative relative expression of COX-2 mRNA was observed to be significantly higher in CRC tissues as compared with that in adjacent normal colon tissues (p = 0.001; Figure 1). Further, low grade CRC showed higher expression of COX-2 mRNA as compared with high grade CRC. Whereas, no significant change in COX-2 mRNA expression was observed between low stages and high stages of CRC (p= 0.585). Also, there was no significant correlation between COX-2 expression and other indices which included age, tumor site, TNM stage, lymph node status, histological type and histological grade (Table 2). However, female CRC patients showed significantly higher (p<0.009) expression of COX-2 than male CRC patients (Figure 2).

*Protein Expression of COX-2*

**Figure 3:**
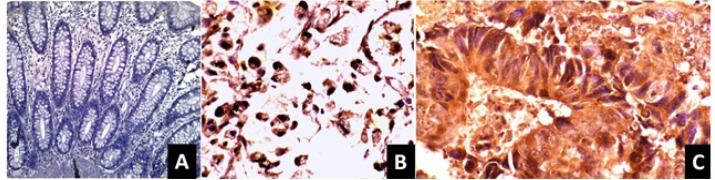
Immunohistochemical Staining of Cyclooxygenase-2 (COX-2): (a) Normal mucosa showing no positivity; (X10) (b) tumor showing moderate cytoplasmic staining (X20); (C) tumor showing strong cytoplasmic staining (X 40)

COX-2 immunostaining were positive in (70%) cases and negative in 30% of cases. Immunohistochemical analysis demonstrated that COX-2 protein was located mainly in the cytoplasm of the tumor tissue. However, normal adjacent colonic epithelium exhibited no expression of COX-2 (Figure 3).

## Discussion

Colorectal cancer is a multi-factorial disease that affects various individuals worldwide. COX-2 represents an important molecular target in CRC prevention and treatment. Nuclear factor kappa B (NF-kB) is a key regulator of COX-2. Recently, we have observed increased expression of NF-kB-p65 in malignant colorectal epithelial cells (Negi et al., 2017). Hence, the present study was undertaken to evaluate the levels of COX-2 in human colonic adenocarcinoma. In the present study, the quantifiable relative expression of COX-2 mRNA was observed to be significantly higher in tissues of CRC patients as compared to their adjacent normal colon mucosa. The precise mechanism of COX-2 over-expression at molecular level is still unknown.

However, numerous studies have observed that cells that over-express COX-2 help in controlling the process of angiogenesis, cause inhibition of apoptosis or increase invasiveness by secreting inflammatory prostaglandins (Tsujii et al., 1998; Cianchi et al., 2001). Certain studies have described that some of the COX-2 inhibitors prevent the recurrence of adenoma among patients who had an initial history of adenoma. They have also observed that COX-2 expression is raised in colorectal adenomas and cancers. All these observations do suggest that there is an important role of COX-2 over-expression in colorectal carcinogenesis (Sheehan et al., 1999; Bertagnolli et al., 2006; Arber et al., 2006). However, the role of COX-2 over-expression in understanding the biologic behaviour of colorectal cancer is still debatable. Since, COX-2 was first demonstrated to be increased in colorectal cancer by Eberhart et al., (1994) and thereafter, numerous studies have shown inconstant expression levels of COX-2 in colorectal cancer (Soumaoro et al., 2004; Yamauchi et al., 2002; Xiong et al., 2005). These inconsistencies in results may be interrelated to detection methods used, patient cohorts, and especially to grading system used in the studies (Fux et al., 2005; Ogino et al., 2008; Yamac et al., 2005). A recent study from North India also showed a high expression of COX-2 in CRC patients but this study has analyzed the COX-2 expression by using Immunohistochemistry (Arundhati, 2017). 

However, in our study, we simultaneously investigated COX-2 expression at both mRNA and protein levels in human colorectal adenocarcinomas using quantitative real-time PCR and immunohistochemistry. Moreover, in the present study, we also correlated the expression of COX-2 mRNA with the clinicopathological features. However, we did not observe any relationship between COX-2 over-expression and clinicopathological features of colorectal cancer patients, though, the gender was significantly related with COX-2 expression. The greater expression was associated with female patients of CRC. This is the foremost study which has observed a signiﬁcant association between COX-2 mRNA over-expression with gender of CRC. Various studies have described the association amid the COX-2 over-expression and the clinicopathological parameters in colorectal adenocarcinoma but the same has not been substantiated in other studies. Our observations are in agreement with the findings of Wu et al., (2003). They observed that COX-2 has a key role to play in the process of colorectal carcinogenesis and it is associated with tumor biological characteristics and patients’ prognosis. However, they did not observe any association between clinicopathological parameters and expression of COX-2. The results of the present study are also supported by the observation of Mahmoud et al., (2014), who have found no association between COX-2 expression and the clinicopathological features except for association between COX-2 expression and gender. Contradictory to results of present study, COX-2 expression was significantly linked with the depth of tumor invasion in another study (Lim et al., 2007). Likewise, Zhang and Sun, (2002) reported that over-expression of COX-2 was well associated with advanced stage of dukes’ classification and colonic site of colorectal cancer. Outcomes of the studies by various other authors have also shown significant relationship between COX-2 expression and various clinicopathological features (Soumaoro et al., 2002; Ogino et al., 2008; Yamac et al., 2005; Castells et al., 2006; Lim et al., 2008). Furthermore, study by Elzagheid et al., (2013) observed that elevated levels of COX-2 expression were significantly related to higher Dukes’ stage and TNM class but in contradictory to our results they found no significant association with gender. Recently, Venkatachala and Rajendran, (2017) reported that COX-2 overexpression was related to increasing stage and depth of invasion. However, there was no significant relationship between elevated COX-2 expression and clinicopathological features including tumor location, age, TNM stage, histopathological classification, lymph node metastasis, and histopathological differentiation which were in accordance with our results.

This is the first study to assess the COX-2 overexpression and its association with clinicopathological features of CRC using quantitative Real-time PCR. Except for one study (Einspahr et al., 2003), in which the COX-2 over-expression in colorectal adenoma was demonstrated by Real-time PCR, all other studies analyzed expression of COX-2 and its association with clinicopathological features of CRC using Immunohistochemistry. The main advantage of real-time PCR is the fact that it is a more quantitative and more sensitive technique as compared with other high-throughput assays. The present study revealed over expression of COX-2 in colorectal carcinoma patients and observed no relationship between mRNA expression of COX-2 and clinicopathological parameters. Further, immunohistochemistry of COX-2 confirmed the quantitative Real time-PCR findings. Moreover, in the present study, early stage tumors showed higher COX-2 mRNA expression in comparison to advanced stage tumors in colorectal carcinoma patients, thus, suggesting its use as a potential marker of early progression of the disease, though the expression was even high at all stages. Currently, there is no such work has been published in which COX-2 expression is studied along with clinicopathological features in colorectal carcinoma using Real-time PCR. Further, presence of COX-2 mRNA over expression in colorectal carcinoma in contrast with normal mucosa suggests that COX-2 does play an important part in cell proliferation in carcinogenesis.

In conclusion, COX-2 expression was found to have a significant association with gender and is assumed to play an important role in colorectal carcinogenesis. Further, these findings support the view that over-expression of COX-2 in colorectal carcinoma suggests its role as a potential biomarker for diagnosis of colorectal cancer with an emphasis on development of COX-2 inhibitors as potential promising chemo-preventive drugs for colorectal cancer.

## Funding

Indian Council of Medical Research (ICMR), New Delhi and DST-PURSE (Government of India) provided the funding for this work.

## Research involving Human Participants

All procedures performed in studies involving human participants were in accordance with the ethical standards of the institutional research committee and with the 1964 Helsinki declaration and its later amendments or comparable ethical standards.

## Informed Consent

Informed consent was obtained from all individual participants included in the study.

## Conflict of Interest

The authors declare that they have no conflict of interest.
